# Embryonic Development and Potential Causes of Reproductive Failure in *Tilia taishanensis* S. B. Liang

**DOI:** 10.3390/plants15182882

**Published:** 2026-09-21

**Authors:** Hanlin Liu, Meixuan Wang, Weihao Song, Xiaoshuo Jiang, Yaxuan Wang, Ruichen Shan, Qian Zhang, Ruixue Wang, Limin Sun

**Affiliations:** 1 Key Laboratory of National Forestry and Grassland Administration for Warm Temperate Forest Ecosystem Conservation and Restoration, Forestry College of Shandong Agricultural University, Tai’an 271018, China; 2 Shandong Academy of Forestry, Jinan 250014, China; 3 Shandong Provincial Forestry Protection and Development Service Center, Jinan 250014, China

**Keywords:** *Tilia taishanensis* S. B. Liang, embryonic development, microspore and megaspore development, male and female gametophytes

## Abstract

In this study, the rare and endangered tree species *Tilia taishanensis* S. B. Liang was selected as the research material, and its embryonic developmental characteristics and abortion mechanism were systematically observed using scanning electron microscopy and resin semi-thin sectioning techniques. The results showed that its pollen was bicellular, the anther wall belonged to the dicotyledonous type with a secretory tapetum, and asynchronous meiosis occurred during microsporogenesis. The pistil possessed a pentalocular ovary and anatropous ovules, and the embryo sac exhibited a typical seven-celled, eight-nucleate structure of the Polygonum type. Male gametophyte development initiated 1–2 weeks earlier than female gametophyte development, although both gametophytes matured synchronously in late June, and the embryo and endosperm developed following the Solanad type and nuclear type, respectively. Multiple reproductive barriers existed in this species: the pollen viability was only approximately 10%, and abnormal tapetal degeneration interrupted nutrient supply for microspores; continuous embryo sac degeneration may contribute to a hollow seed rate of 90%; developmental asynchrony between male and female gametophytes further reduced fertilization efficiency. This study explores the embryonic developmental characteristics and abortion mechanism of *T. taishanensis*, providing cytological evidence for elucidating its endangered causes and germplasm conservation.

## 1. Introduction

*Tilia taishanensis* S. B. Liang is a new species of the *Tiliaceae* family and the *Tilia* genus, first discovered and named by Liang Shubin on Mount Tai [1]. It is a tree species endemic to China, with a height of up to 25 m. The bark is gray with shallow vertical fissures, and the leaves are nearly round or broadly ovate. The flowers are pale yellow and arranged in cymose inflorescences. The fruit is inverted-ovoid or nearly spherical. The flowering period lasts from June to July, for approximately 15 days, and the fruit ripens in late September. This species is a heliophilous deep-rooted tree that prefers sunlight and fertile, well-drained soils. It has strong cold resistance but is intolerant of waterlogging [2]. The seeds require artificial pre-germination to sprout, and young trees are shade-tolerant, while mature trees need full sunlight. This species is distinguished from other *Tilia* species by its sessile bracts [3]. It is found in limited quantities in Shandong and holds extremely high value [4]. The wood can be used in construction [5], musical instruments, and other industries. Its aesthetic tree shape makes it suitable for use as a street tree, and it is also a high-quality honey source [6]. The honey produced from it is considered top-grade.

Research on the floral organ development of *Tilia* species has mostly focused on *Tilia sinensis* [7], *Tilia platyphyllos* [8], and others, with basic characteristics such as four-lobed anthers, three-grooved pollen, and polygonal-type embryo sacs already identified [9]. However, the developmental timing of pollen and ovule, as well as the regulatory mechanisms behind the programmed degeneration of the endothecium, remain insufficiently analyzed. In terms of seed formation, it is known that the genus commonly exhibits nuclear-type endosperm free-cell development, solanaceous-type embryo development, and physiological embryo drop. However, the exact abortive stages that lead to the formation of empty seeds have yet to be elucidated. The primary propagation techniques are cuttings and grafting, while tissue culture systems are still underdeveloped [10]. Seed propagation is limited by the extremely low germination rate [11,12]. *T. taishanensis,* as an endemic and extremely small population in Shandong, has only been described morphologically in earlier studies. Its pollen viability is about 10%, and the seed emptiness rate is as high as 90%. Pollen abortion and embryo sac degeneration may be critical factors contributing to its endangered status.

The present study aims to characterize the microsporogenesis, megasporogenesis, male- and female-gametophyte development, embryogenesis and endosperm formation of *T. taishanensis* via histological observations. We also document a series of developmental anomalies that may be linked to its poor reproductive performance, so as to provide embryological basis for further conservation research on this narrowly distributed endemic species.

## 2. Materials and Methods

### 2.1. Experimental Site Overview

The experiment was conducted at Panguan Ridge of Mount Tai in Tai’an City, Shandong Province, China, at an altitude of 600 m. This region features a temperate semi-humid continental monsoon climate with distinct four seasons, synchronized light and temperature conditions, and coincident rainfall and heat periods. The average temperature in summer is approximately 20 °C to 23 °C, with an average relative humidity of 80% to 85%. In winter, the average temperature is around −5 °C to −2 °C, with an average relative humidity of 60% to 70%.

### 2.2. Experimental Materials

The material of *T. taishanensis* used in this study was collected from a 200-year-old ancient tree at Panguanling, Mount Tai, at an altitude of 600 m. This is the first-discovered individual of *T. taishanensis*, with a height of 13.0 m, a trunk diameter at breast height (DBH) of 63.7 cm, and a crown spread of 16.0 m (east–west) and 13.0 m (north–south). The tree flowers and fruits abundantly; its flowering period lasts from late June to early July, but the seed empty rate is as high as 90%. Multi-year field surveys have not identified any additional adult wild individuals within Mount Tai and its surrounding potential habitats, and this tree represents the only confirmed wild mature individual of this narrowly endemic species. This single-individual sampling constitutes a limitation of the present study; the developmental characteristics and reproductive defects documented here should therefore be interpreted as representative of this investigated individual and local population, and caution should be exercised when generalizing these findings to the species as a whole.

### 2.3. Research Methods

#### 2.3.1. Sample Collection

In early July, mature but unopened anthers of *T. taishanensis* were collected exclusively from the investigated wild-mature tree growing in the Panguanling area of Mount Taishan, with filaments removed, for scanning electron microscopy observation. Samples were collected 25 times at 1-day intervals, covering flower buds and young flowers of different developmental stages for resin semi-thin section preparation. For each sampling, 25–30 flowers were collected; multiple anthers per flower were used for cytological observation, and ovaries were dissected to isolate ovules. A total of 200 ovules were embedded and sectioned to yield 240 histological slices. Serial sections from one ovule were treated as non-independent observations to avoid pseudo-replication.

#### 2.3.2. Scanning Electron Microscopy (SEM)

The collected anthers were prefixed with 2.5% glutaraldehyde and post-fixed with 1% osmium tetroxide, followed by three washes with 0.1 mol/L phosphate buffer. The samples were then dehydrated using an ethanol series, transitioned with isoamyl acetate, and critical-point dried with CO_2_. The anthers were mounted on stubs, and the pollen was gently removed. After gold sputter-coating, the samples were observed and photographed using a JSM-6490LV scanning electron microscope (JEOL, Tokyo, Japan) equipped with a built-in digital imaging camera [13].

#### 2.3.3. Preparation of Resin Semi-Thin Sections

After the samples were prefixed with 2.5% glutaraldehyde, post-fixed with 1% osmium tetroxide, and washed with phosphate buffer, they were dehydrated through a graded ethanol series from 20% to 100%, followed by infiltration with JB4 resin for two cycles of 3 days each. The specimens were then embedded in JB-4 resin mixture and polymerized at 65 °C for 2 h in an oven. After trimming the resin blocks, 2 μm thick sections were cut using a microtome, water-dispersed, dried, and stained with toluidine blue. The sections were observed and photographed under an Olympus BX51 microscope (Olympus, Tokyo, Japan) equipped with an Olympus DP72 digital camera (Olympus, Tokyo, Japan) for analysis [14].

An embryo sac was regarded as degenerated when showing obvious cytoplasm shrinkage, nuclear disintegration, and disorganized cell structure under semi-thin sections.

#### 2.3.4. Pollen Viability Assessment

Pollen viability was assessed using the I_2_-KI histochemical staining method. Anthers were harvested from mature non-dehisced flower buds 1–2 days before anthesis. Pollen grains were examined under light microscopy in multiple random fields. Pollen with densely stained cytoplasm was considered viable, while shrunken, empty or weakly stained grains were classified as non-viable. Repeated microscopic observations indicated that pollen viability of *T. taishanensis* was approximately 10% [15].

#### 2.3.5. Seed Empty Rate Estimation

The empty seed rate of approximately 90% was estimated based on the proportion of ovules with degenerated embryo sacs among the 200 sectioned ovules. Since embryo sac degeneration prevents normal embryo formation, the degeneration frequency observed in histological sections was used as an indirect estimate of the empty seed rate [16].

## 3. Results

### 3.1. The Development of Microspores and the Male Gametophyte

#### 3.1.1. Pollen Morphology

**Figure 1:**
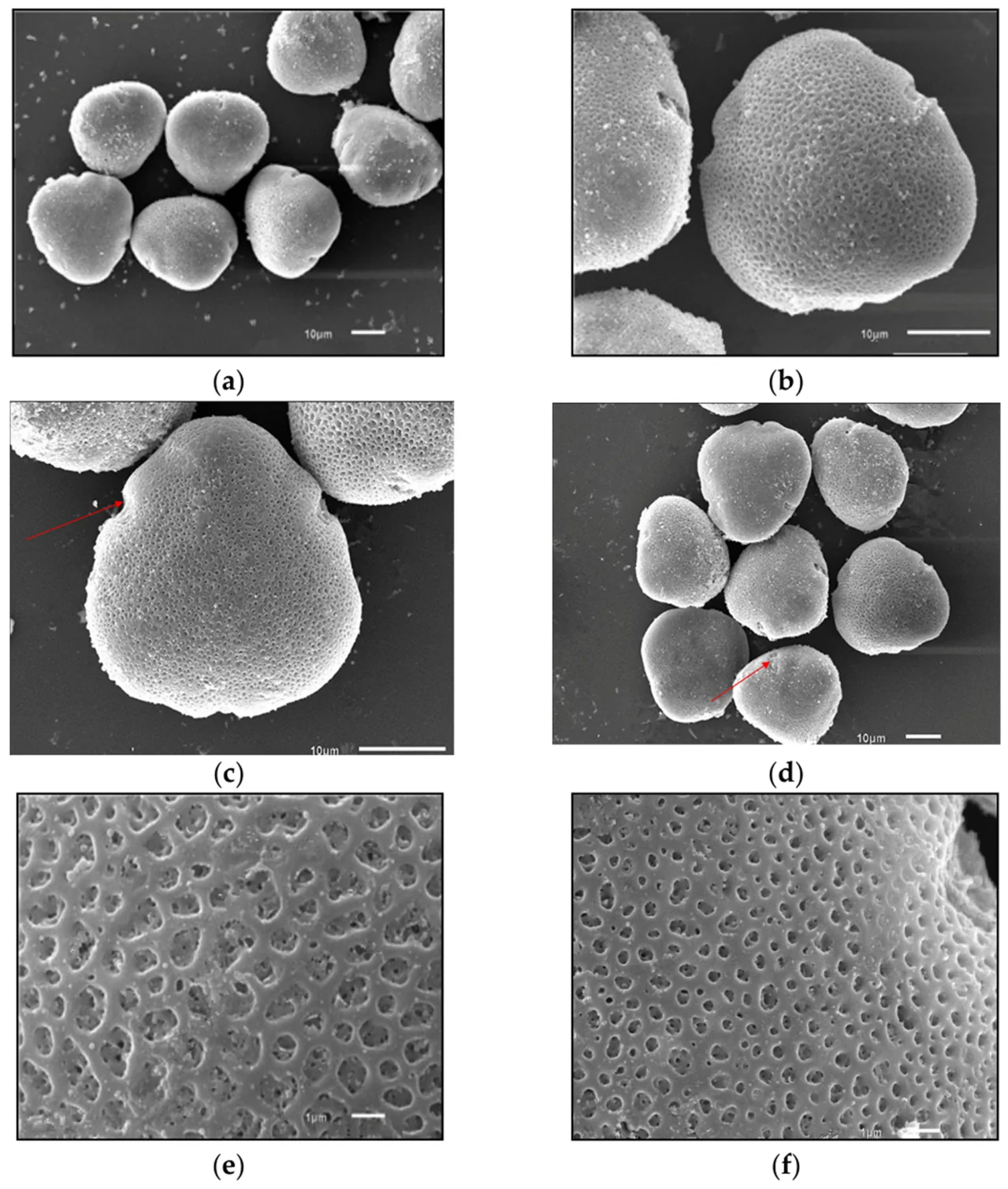
Pollen morphology of *T. taishanensis* under SEM. (**a**) Pollen population view (×1000). (**b**) Polar view of pollen grain (×2500). (**c**) Germination colpus on pollen surface (×2500). (**d**) Germination pore with thickened inner margin (×1000). (**e**,**f**) Exine ornamentation with cavernous patterns (×10,000). Red arrows indicate the germination colpi (**c**) and pore (**d**).

The size of the pollen of *T. taishanensis* is approximately 31.08 (29.38–31.25) × 22.37 (17.5–23.13) μm. Under scanning electron microscopy, the polar view of the pollen is triangular (Figure 1a,d), while the equatorial view is mostly elliptical. The pollen has three germination grooves along the polar axis (Figure 1c), which are approximately evenly spaced. The pores are short and deep, with the inner edge of the grooves significantly thickened, and slight protrusions on both sides (Figure 1b). The pore membranes are sunken rather than protruding. The surface of the pollen is sculpted with cavernous patterns (Figure 1e), densely covered with punctate pits and a few granular protrusions. The cavities are multilayered, unevenly distributed, and vary in size, with irregular circular or elliptical shapes. The density of the mesh near the polar view is lower than that near the equatorial view, and the mesh gradually becomes smaller near the germination grooves (Figure 1f).

#### 3.1.2. Development of the Anther Wall

**Figure 2:**
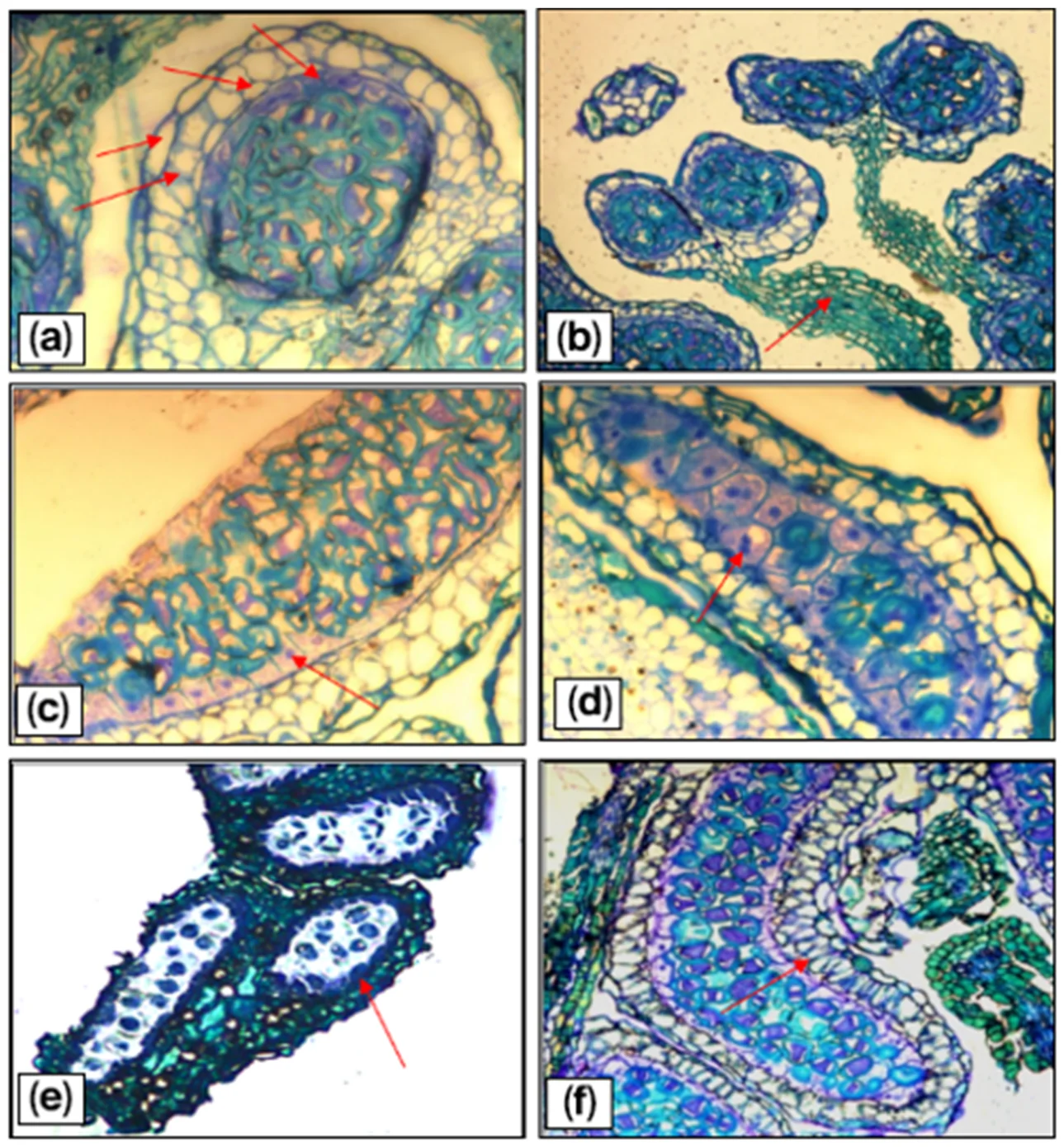
Anther wall and tapetum development in *T. taishanensis*. (**a**) Four-layer structure of the anther wall (×400). (**b**) Vascular bundle in the filament (×200). (**c**) Uninucleate tapetal cell (×400). (**d**) Binucleate tapetal cells (×400). (**e**) Tapetal cells with abundant cytoplasmic content (×200). (**f**) Band-like thickening of the endothelial inner tangential wall (×400). Red arrows indicate the four-layer anther wall structure (**a**), vascular bundle (**b**), tapetal cells (**c**–**e**), and endothelial wall thickening (**f**).

After the anther becomes a less distinct tetragonal shape, large sporogenous cells with large nuclei, dense cytoplasm, and a longitudinal arrangement differentiate beneath the epidermal cells at the corners. At this stage, a large number of longitudinal conducting cells from the attached filament transport nutrients to the anther (Figure 2b). The sporogenous cells undergo periclinal and anticlinal divisions to produce primary wall cells and primary microsporocytes. The former further divides to form a concentric cell layer that, together with the outer epidermis, constitutes the anther wall, while the latter undergoes mitosis to develop into secondary microsporocytes, which then give rise to the microspore mother cells. At this stage, the anther wall consists of four layers, from outside to inside: the epidermis, the endothecium, the middle layer, and the tapetum (Figure 2a). Its development follows the dicotyledonous type of anther wall formation as classified by Davis among the four types of angiosperm anther wall formation (Figure 2).

It should be noted that only the number of tapetal nuclei (uninucleate and binucleate cells) was observed from semi-thin sections in this study, and the actual ploidy level of these nuclei was not determined. Binucleate tapetal cells in many angiosperms are commonly generated by endomitosis, which leads to nuclear polyploidization within a single tapetal cell.

The epidermis consists of the earliest-formed single-layer cuticular cells, which only divide periclinally to accommodate internal growth. As the anther grows, cell morphology changes, and during pollen dispersal, the nuclei of the cells degrade, staining deepens, intercellular spaces widen and nearly disintegrate, leaving only traces of desiccation. The endothecium (fibrous layer) consists of a single layer of organized cells and reaches its maximum development at maturity. The outer tangential walls are thin, while the inner tangential walls thicken in a band-like manner (Figure 2f). At the junction of the two pollen sacs on the same side, the cell walls remain thin, facilitating the dehiscence of the pollen sacs for pollen release. At the junction of the two pollen sacs on the same side, the cell walls remain thin, facilitating the dehiscence of the pollen sacs for pollen release. The middle layer consists of only one layer of cells. During the meiotic division of the microspore mother cells, they are compressed and become flattened. The mononucleate stage begins to disintegrate at the periphery, and it completely disappears by the bicellular pollen stage. The tapetum, as the innermost layer of cells, is derived from the primary wall layer. After the formation of the microspore mother cells, tapetal cells gradually enlarge and the protoplast increases. Initially, tapetal cells are uninucleate (Figure 2c) and develop into binucleate cells (Figure 2d). The tapetum reaches its developmental peak during meiosis of the microspore mother cells. Tapetal cells continue to supply nutrients during microspore development, with their cytoplasmic content gradually diminishing (Figure 2e). When the pollen matures, nutrient secretion is complete, and the tapetal cells disintegrate in situ, characteristic of a secretory (glandular) tapetum.

#### 3.1.3. The Correlation Between the Development of the Anther Wall and Pollen Development

After cell division, the primary wall cells and the outer epidermis form the anther wall. As the secondary microsporocytes develop into microspore mother cells, the anther wall differentiates into a four-layer structure. During the meiosis of the microspore mother cells, the middle layer cells become flattened, gradually disintegrate, and are absorbed. The endothecium cells contain abundant protoplasm, with dense cytoplasm and large cell volume. As the anther develops, the cytoplasmic content of tapetal cells decreases continuously. When the pollen matures, the tapetal cells disintegrate in situ, and the tapetum degenerates, leaving only the cell wall structure.

**Figure 3:**
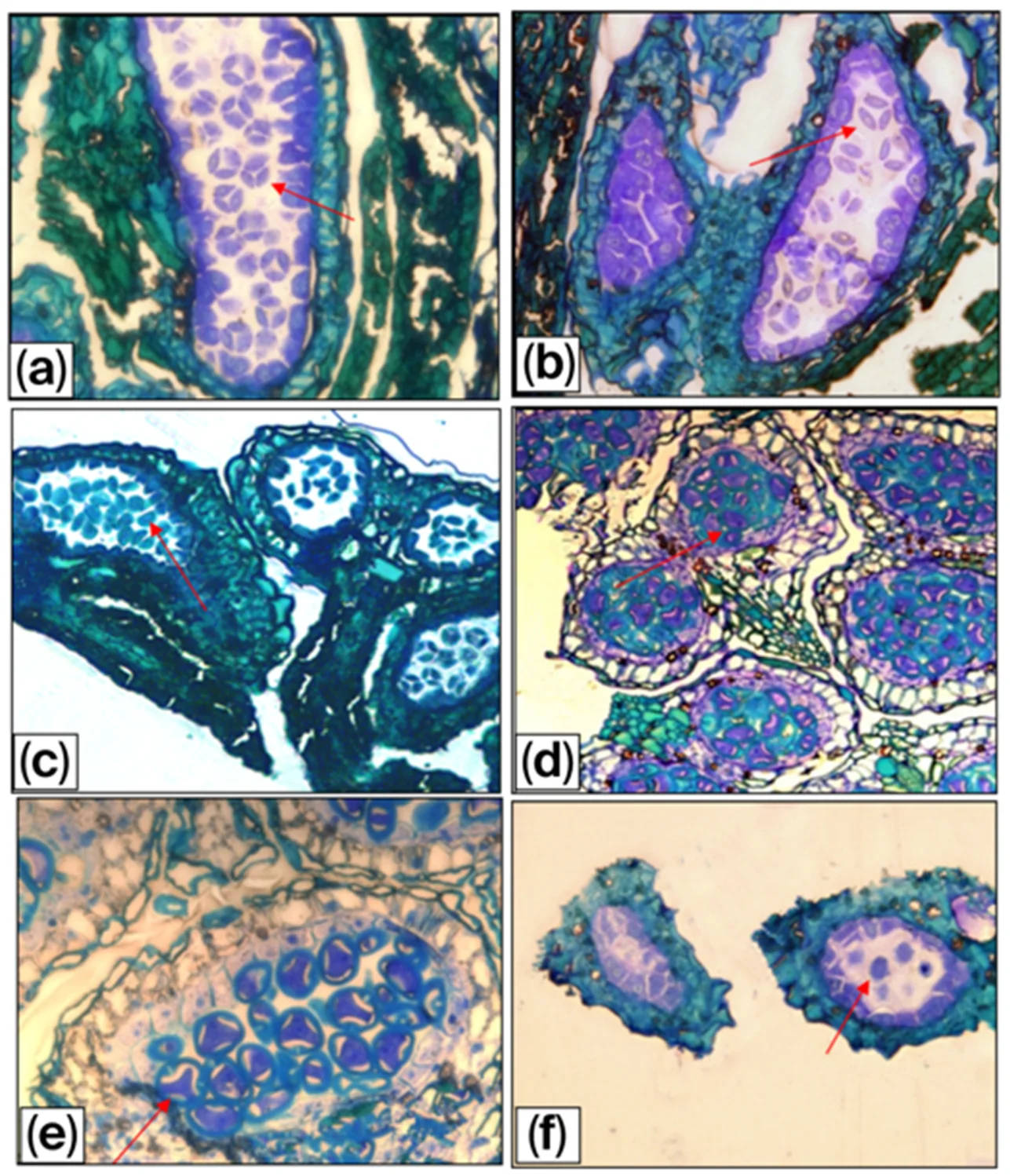
Microsporogenesis and male gametophyte development in *T. taishanensis*. (**a**) Microspore tetrad (×400). (**b**) Callose walls separating microspores within the tetrad (×400). (**c**) Newly released microspore (×200). (**d**) Uninucleate microspore (×200). (**e**) Mid-to-late uninucleate microspore with peripheral nucleus (×400). (**f**) Two-celled pollen grain (×200). Red arrows indicate the microspore tetrad (**a**), callose wall (**b**), newly released microspore (**c**), uninucleate microspore (**d**), peripheral nucleus (**e**), and the generative cell (**f**).

Tashan linden has a cymose inflorescence with bisexual flowers, where the development of the stamens precedes that of the pistils. The development of the microspores and male gametophytes occurs before that of the megaspores and female gametophytes. The stamen primordium develops into the filament and anther. Initially, the anther is nearly quadrangular in shape, with the epidermis covering the periphery. In late May, during the initial formation of the pollen sacs, thin-walled cells beneath the epidermis at the corners differentiate into sporogenous cells. These cells undergo mitosis to form primary microsporocytes, which then divide to form secondary microsporocytes, and by early June, they develop into microspore mother cells. The microspore mother cells are large, with large nuclei and dense protoplasm, and no noticeable vacuole. Later, they form a callose wall that thickens, with the cells becoming spherical and separated, entering the meiotic division stage. The first meiotic division undergoes multiple stages and is time-consuming. After division, a binucleate cell is formed, which then undergoes the second meiotic division to form a tetrahedral tetrad of microspores (Figure 3a). The four microspores are surrounded by a shared callose wall and are spaced apart (Figure 3b). Subsequently, the callose wall disintegrates, and the microspores are released into the anther locule. The meiosis of microspore mother cells is asynchronous. The outer anther of the same flower undergoes meiosis earlier than the inner anther. Within the same anther, different locules and even different stages of division coexist.

The recently released microspores (Figure 3c) undergo a brief contraction period before elongating longitudinally into an elliptical shape, becoming mononucleate microspores (Figure 3d). After absorbing nutrients, the microspores increase in size, with the cytoplasm becoming vacuolated to form a large central vacuole. The nucleus is pushed to one side of the cell, entering the mononucleate periphery stage (Figure 3e). During the mononucleate peripheral stage, the microspore undergoes unequal mitotic division to produce a vegetative cell and a generative cell, which are markedly different in size. The generative cell eventually detaches and lies freely within the cytoplasm of the vegetative cell, forming binucleate pollen (Figure 3f). As the bicellular pollen further matures, the large vacuole disappears.

### 3.2. Megaspore Formation and the Development of the Female Gametophyte

#### 3.2.1. Style, Stigma, Ovary, and Ovule

**Figure 4:**
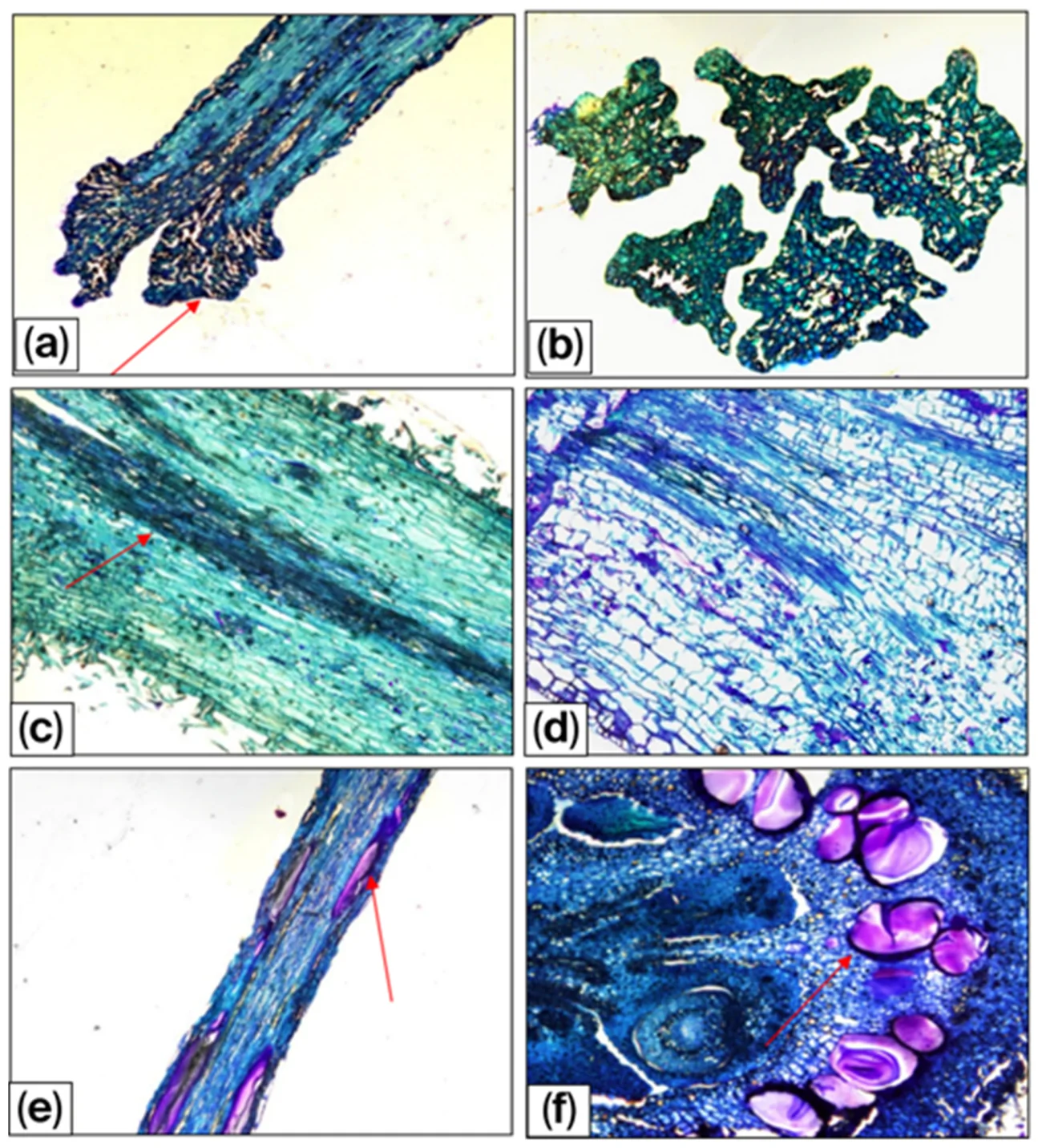
Stigma, style and ovary structure of *T. taishanensis*. (**a**) Five-lobed stigma (×200). (**b**) Stigma lobe with large, densely cytoplasmic cells (×800). (**c**) Spiral tracheids in the style (×400). (**d**) Transmitting tissue in the center of the style (×800). (**e**) Secretory cavities on both sides of the stylar canal (×200). (**f**) Secretory cavities within the ovary wall (×200). Red arrows indicate the stigma lobes (**a**), spiral tracheids (**c**), and secretory cavities (**e**,**f**).

The stigma of Tashan linden is 5-lobed, with large cells and dense cytoplasm (Figure 4a,b). The style is of the closed type, connecting the stigma and ovary. The style canal lacks a hollow structure, and the central tissue is composed of a group of narrow, thin-walled cells with indistinct nuclei, sparse cytoplasm, and abundant intercellular strands, which serve as guiding cells. The pollen tube passes through this guiding tissue to reach the ovary. Numerous vascular bundles in the form of spiral tracheids are distributed within the style (Figure 4c,d). On both sides of the style canal, there are also large, elongated cavities filled with lightly stained material, which may represent secretory structures (Figure 4e). Furthermore, the epidermis of the style is not covered by any trichomes.

**Figure 5:**
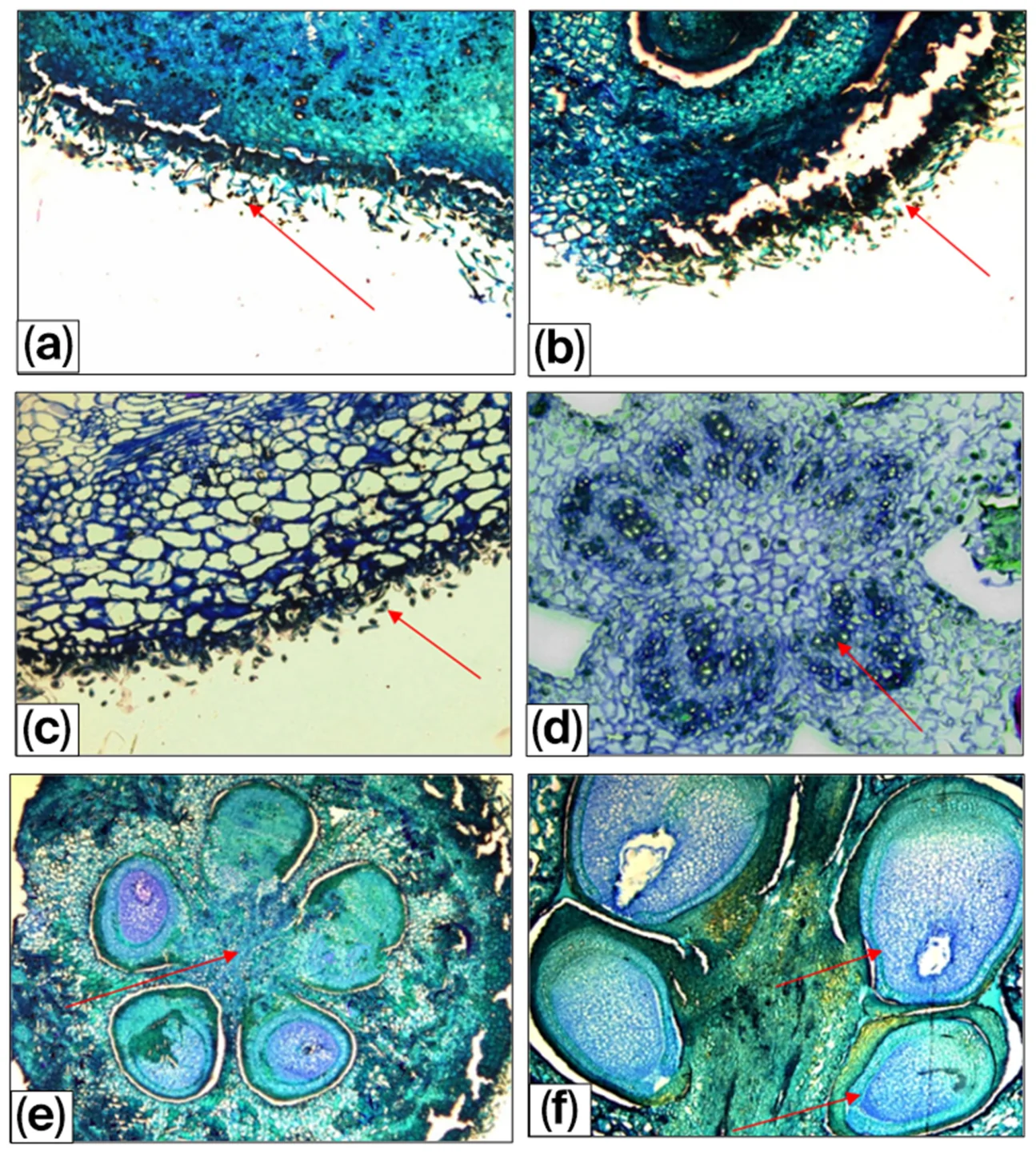
Differentiation of T-shaped trichomes and ovule arrangement in *T. taishanensis*. (**a**) T-shaped trichomes distributed on the outer ovary wall (×400). (**b**) Three-celled initial stage of a trichome, showing the intermediate cell with reduced cytoplasm and degenerating nucleus (×400). (**c**) Two elongated lateral cells with thickened cell walls forming the T-shaped structure after complete degeneration of the intermediate cell (×800). (**d**) Vascular bundles in the ovary wall (×800). (**e**) Ovules arranged along the central axis (×200). (**f**) Two ovules per locule (×200). Red arrows indicate T-shaped trichomes (**a**), the intermediate cell undergoing degeneration (**b**), the two lateral cells forming the T-shape (**c**), vascular bundles (**d**), and ovules (**e**,**f**).

*T. taishanensis* lacks an ovary stalk. The ovary wall is divided into an inner wall and an outer wall. The cells near the outer wall of the inner ovary are large, with large nuclei, dense cytoplasm, and small vacuoles. The inner wall consists of multiple layers of cells, with the inner cells being smaller, having smaller nuclei, larger vacuoles, and containing cavities filled with lightly stained material that may represent secretory products (Figure 4f). The outer wall of the ovary consists of fewer layers of larger cells, with vacuoles occupying almost the entire cell. The nuclei are small, and the cytoplasm is sparse. The outer wall is densely covered with numerous “T”-shaped trichomes (Figure 5a). These trichomes initially consist of three cells. As the ovary develops, the middle cell’s nucleus becomes smaller and less distinct, cytoplasm decreases, vacuoles disappear, and the cell disintegrates (Figure 5b), leaving only the cell wall structure. The two lateral cells elongate longitudinally, with smaller nuclei and thickened cell walls, ultimately forming the “T”-shaped structure (Figure 5c). The ovary contains numerous vascular bundles, which facilitate the transport of nutrients and water to the interior through these bundles (Figure 5d).

**Figure 6:**
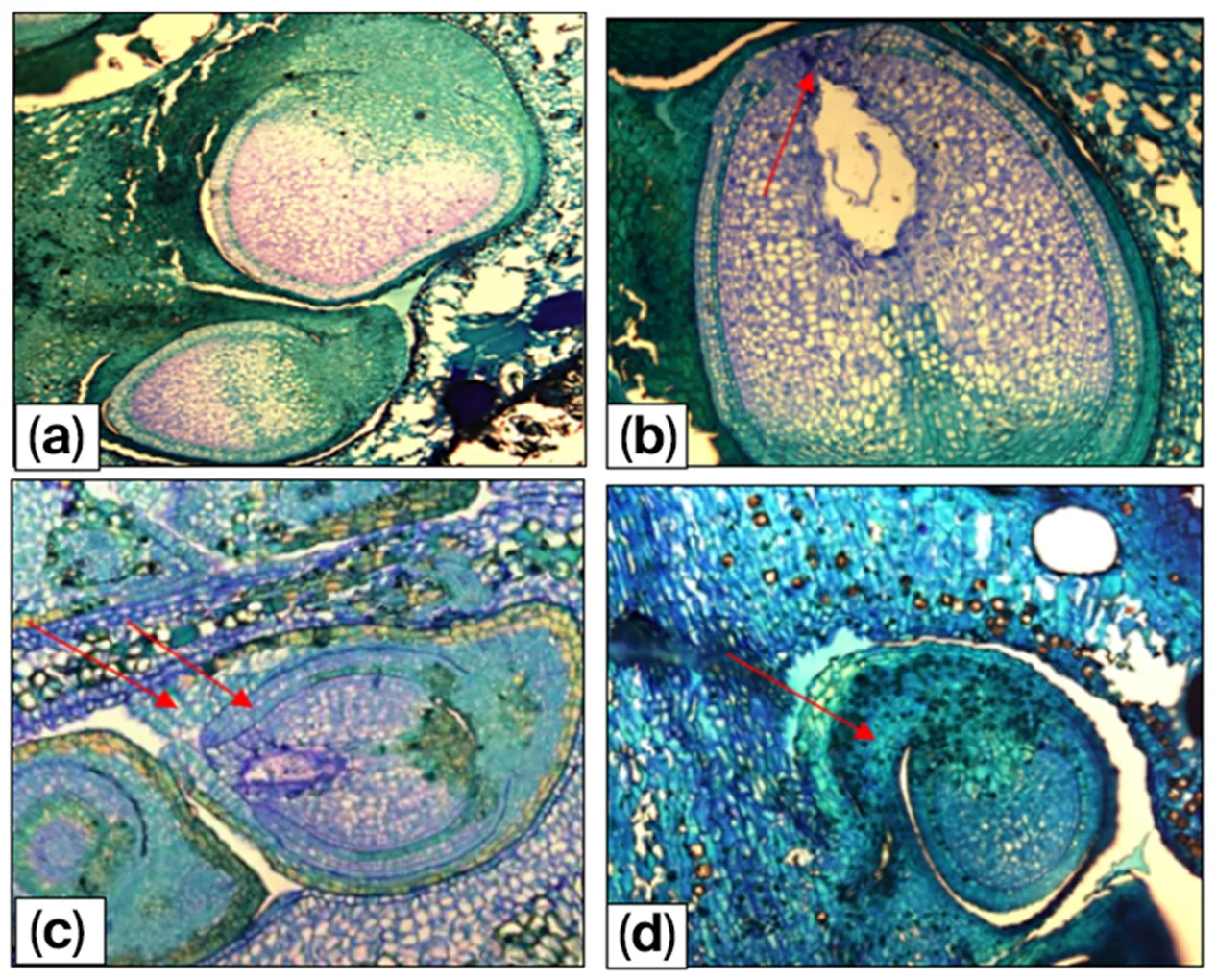
Ovule structure of *T. taishanensis*. (**a**) Anatropous ovule (×200). (**b**) Micropyle formed by the outer integument (×400). (**c**) Bitegmic ovule with distinct inner and outer integuments (×200). (**d**) Funiculus attaching the ovule to the placenta (×200). Red arrows indicate the micropyle (**b**), integuments (**c**), and funiculus (**d**).

The ovary of *T. taishanensis* is superior, consisting of 5 chambers, each containing 2 ovules. It belongs to the type with thick chalazal tissue and double integuments. The ovules are arranged along the central axis within the ovary chambers, on a central placentation (Figure 5e,f), and are anatropous (Figure 6a), composed of the nucellus, integuments, micropyle, and funiculus. During the early stages of pistil development, ovule primordia are formed on the placental tissue along the ventral suture of the carpel. The apical part of the primordium develops into the nucellus, while the basal part develops into the funiculus (Figure 6d). Cells near the nucellus divide more rapidly and grow upwards around the nucellus to form the integuments (Figure 6c). The outer integument grows faster than the inner integument and the nucellus, eventually surrounding both, and encircles the upper part of the nucellus to form the micropyle (Figure 6b). However, in a portion of ovules, integument development was asymmetric: the outer integument on one side elongated markedly more than that on the opposite side, resulting in a tilted or laterally displaced micropyle that was not aligned with the funicular axis. This asymmetric growth pattern was observed across different developmental stages of ovule differentiation.

#### 3.2.2. Megaspore Formation

**Figure 7:**
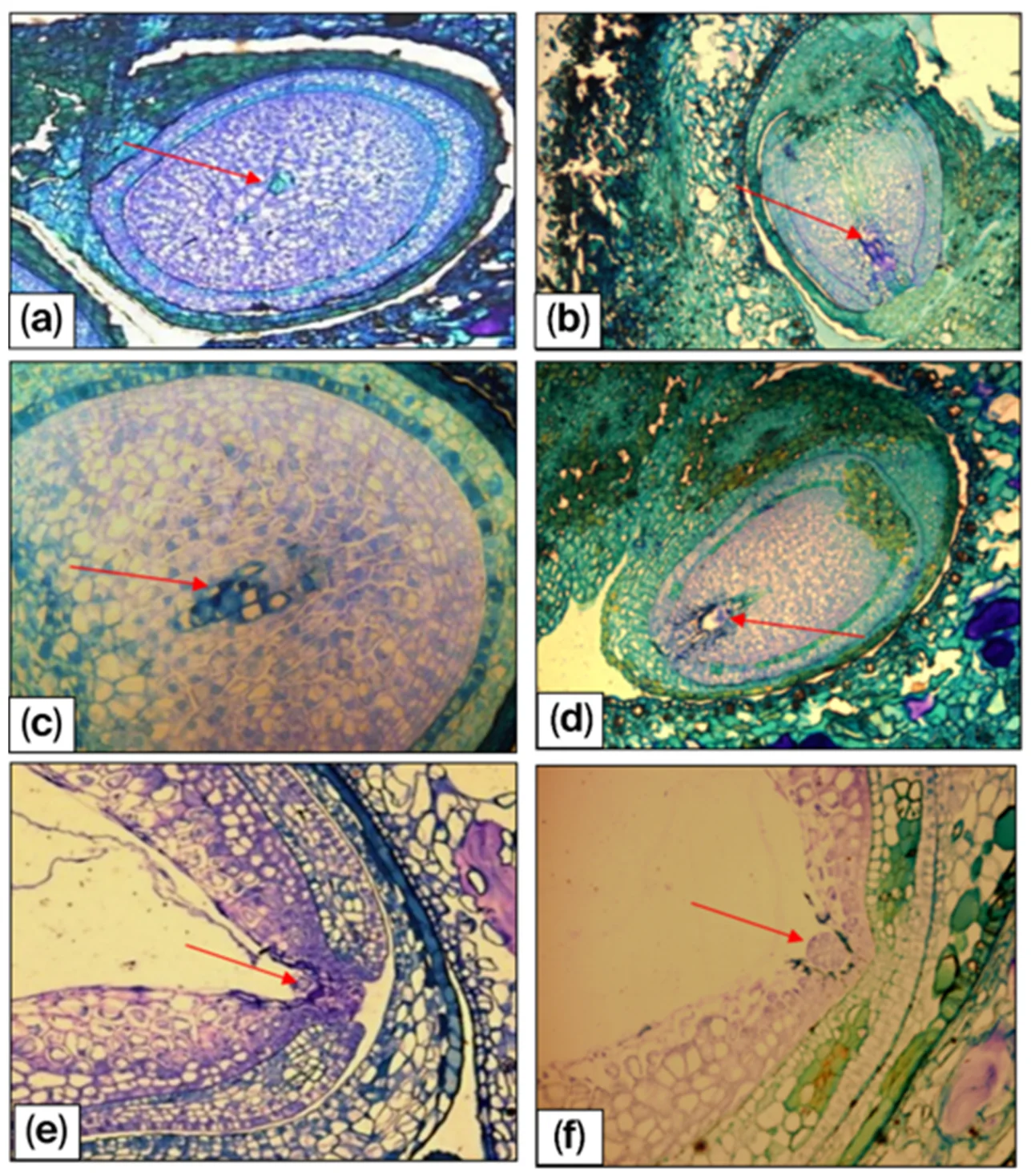
Megasporogenesis, female gametophyte and embryo development in *T. taishanensis*. (**a**) Megaspore mother cell (×200). (**b**) Dyad after the first meiotic division (×200). (**c**) Linear tetrad of four megaspores, with the chalazal megaspore functioning as the functional megaspore (×800). (**d**) Central cell with two unfused polar nuclei in the mature embryo sac (×200). (**e**) Zygote after fertilization, showing the large female nucleolus and smaller male nucleolus (×400). (**f**) Globular embryo with densely cytoplasmic cells (×800). Red arrows indicate the megaspore mother cell (**a**), dyad (**b**), functional megaspore (**c**), central cell (**d**), zygote (**e**), and globular embryo (**f**).

Megaspore formation is the precursor stage of female gametophyte development. Specifically, during the early stages of pistil differentiation, the ovary surface forms ovule primordia. The nucellus is composed of a mass of thin-walled cells, and as the integuments develop, large sporogenous cells with prominent nuclei and dense cytoplasm differentiate beneath the epidermal cells of the nucellus. The sporogenous cells undergo mitosis to produce peripheral cells and megasporocytes. The latter becomes the megaspore mother cell (Figure 7a). The peripheral cells divide to form numerous nucellar cells, which enclose the megaspore mother cell. The megaspore mother cell begins two rounds of meiosis in late June. The first meiotic division progresses through the leptotene, zygotene, pachytene, diplotene, and diakinesis stages. During the interphase, the nucleus is centrally located. In metaphase, the bivalent chromosomes align along the equatorial plate, and in anaphase, the homologous chromosomes are pulled toward opposite poles, ultimately forming dyads (Figure 7b). Subsequently, the second meiotic division results in four linearly arranged megaspores, forming the megaspore tetrad (Figure 7c).

#### 3.2.3. Development of the Female Gametophyte

The development of the embryo sac in *T. taishanensis* begins with the four megaspores formed during meiosis. Only the megaspore near the chalazal end develops into the functional megaspore, while the remaining three megaspores near the micropyle degenerate and disappear. The functional megaspore increases in size and forms a mononucleate embryo sac. It then undergoes three rounds of mitotic division, successively forming a binucleate, tetranucleate, and octonucleate embryo sac. The octonucleate embryo sac enters the cellular differentiation stage to form the mature embryo sac. The mature embryo sac consists of a seven-celled, eight-nucleate structure. Three nuclei at the micropyle end differentiate into the egg apparatus (two synergids and one egg cell), while three nuclei at the chalazal end differentiate into antipodal cells (short-lived and prone to degeneration). The remaining two polar nuclei, one from each end (micropyle and chalazal), move toward the center and fuse to form the central cell, which contains two nuclei (Figure 7d). In the early stages of the mature embryo sac, the central cell is the largest and is almost entirely occupied by a large vacuole. The two cell nuclei are close to each other but have not yet fused, and the nucleolus is prominent. In the later stage, the two nuclei fuse to form a single nucleus. The egg cell, central cell, synergids, and antipodal cells together form the female reproductive unit.

According to classification standards, *T. taishanensis* has a Polygonum-type embryo sac, where the sporogenous cells divide to form peripheral cells and the megasporocyte (megaspore mother cell). The megaspore mother cell undergoes meiosis to form four megaspores, with only the megaspore at the chalazal end developing into the functional megaspore, ultimately forming the mature embryo sac. After the embryo sac matures, the ovule is inverted, and the line connecting the chalazal, nucellus, and micropyle is nearly parallel to the funiculus. The internal cells are fully differentiated. The egg cell, which is the female gamete, is pear-shaped, with the nucleus located at the chalazal end and a large vacuole at the micropyle end. The synergid cells are located at the micropyle end, are inverted-ovoid in shape, with polarity opposite to that of the egg cell, and have a short lifespan. The central cell is located between the egg apparatus and antipodal cells. It is highly vacuolated and contains two unfused polar nuclei. The three antipodal cells are positioned at the chalazal end of the embryo sac. In many angiosperms, antipodal cells participate in nutrient translocation, transporting materials from surrounding nucellar tissues toward the embryo sac to support female-gametophyte development. However, in *T. taishanensis,* these cells degenerate before completing full cellularization, which may weaken nutrient supply for the developing embryo sac.

### 3.3. Comparison of the Development of the Pistil and Stamen

By the time the pollen matures, the mature octonucleate embryo sac has already formed. In late May, the anther has already differentiated to the stage of sporogenous cells, while the ovule is still at the primordium stage. In early June, when the microspore mother cell enters meiosis, sporogenous cells are only produced in the nucellus. When the megaspore mother cell undergoes meiosis, the anther has already formed mononucleate microspores. During the development of the mononucleate microspores into bicellular microspores, the mononucleate embryo sac in the ovule undergoes two rounds of mitosis, forming a tetranucleate embryo sac. By the time the pollen matures, the mature octonucleate embryo sac has already formed. This indicates that the female gametophyte develops more rapidly in the later stages.

### 3.4. Development of the Embryo and Endosperm

#### 3.4.1. Development of the Embryo

The development of the embryo begins with the fusion of the sperm and egg cell to form the zygote. During fertilization, the pollen tube enters the embryo sac through the micropyle, first reaching the synergids, then releasing two sperm cells. One sperm fuses with the egg cell, and the other fuses with the central cell, forming the zygote and fertilized polar nuclei (Figure 7e). The fertilized zygote has a large female nucleolus and a smaller male nucleolus. After a dormant period (characterized by slight reduction in size, cytoplasmic condensation, and reduction in vacuole size), it undergoes transverse, unequal mitotic division, forming the basal cell (near the micropyle end, large with a large vacuole) and the apical cell (near the chalazal end, small with concentrated cytoplasm), forming a two-cell proembryo. During this stage, the fertilized polar nuclei begin dividing to form multiple free nuclei. The two-cell proembryo develops into a three-cell proembryo (with the basal cell undergoing transverse division first, and the apical cell dividing later), then into an inverted T-shaped four-cell proembryo (with the apical cell dividing longitudinally), and further divides mitotically to form a six-cell proembryo. The proembryo stage lasts until organ differentiation. From the developmental behavior, the embryo development of *T. taishanensis* follows a Solanum-type pattern. Cells produced by the division of the basal cells contribute to the formation of the hypocotyl, while cells produced by the division of the apical cells contribute to the formation of the embryo body. The resulting cylindrical embryo further differentiates into a small, dense-protoplasm spherical embryo (Figure 7f).

#### 3.4.2. Development of the Endosperm

**Figure 8:**
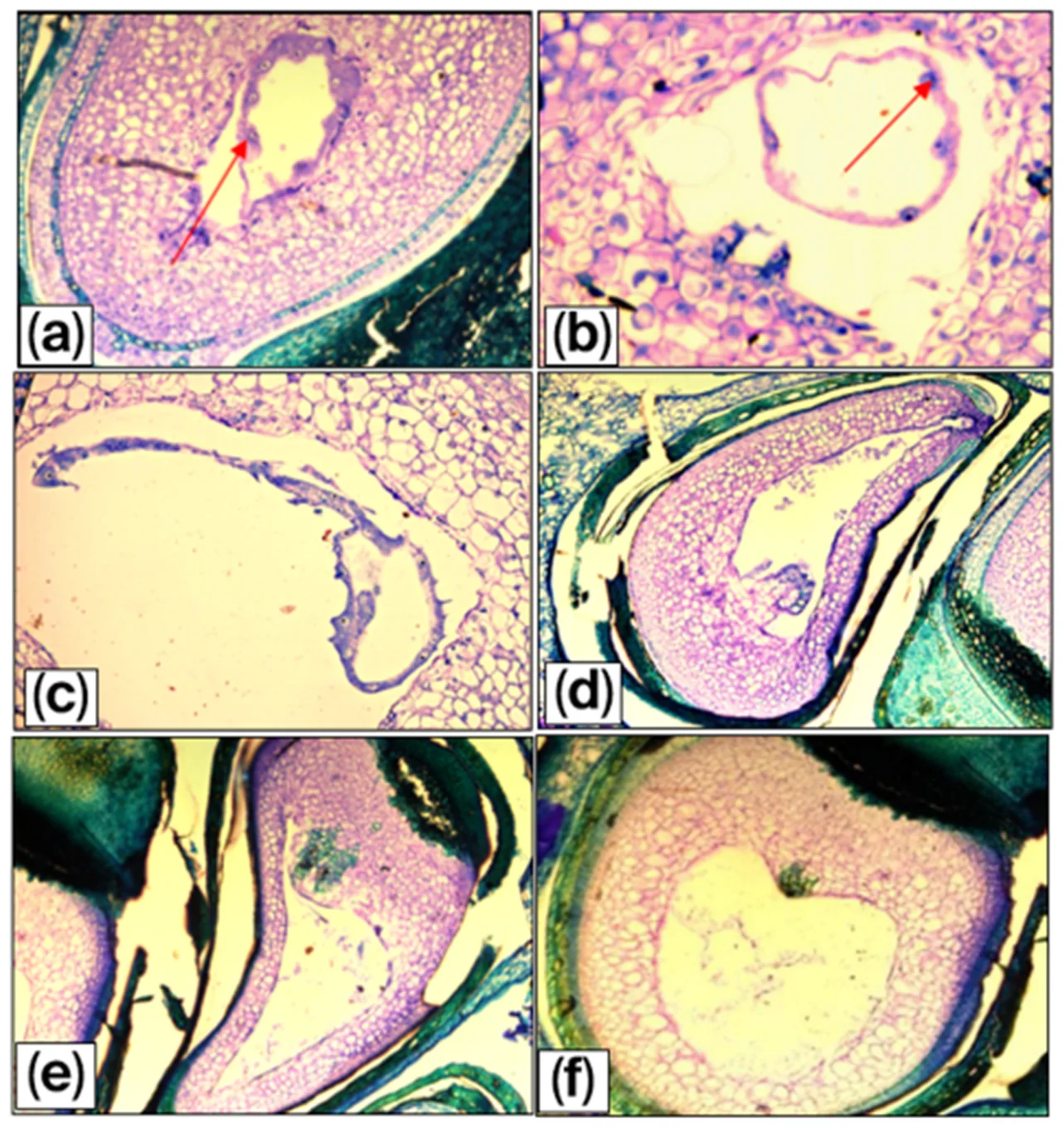
Endosperm development and embryo sac degeneration in *T. taishanensis*. (**a**) Free endosperm nucleus with two nucleoli (×400); (**b**) Free nucleus surrounded by dense cytoplasm (×800); (**c**) Bead-like arrangement of free endosperm nuclei along the embryo sac wall (×800). (**d**) Free endosperm nuclei distributed in the embryo sac (×200). (**e**) Early-stage embryo sac degeneration with cytoplasmic shrinkage (×200). (**f**) Late-stage embryo sac degeneration with disintegrated cell structure and deeply stained remnants (×400). Red arrows indicate free endosperm nuclei (**a**,**b**), and degenerating embryo sac cells (**e**,**f**).

After the sperm fuses with the polar nuclei, the primary endosperm nucleus immediately undergoes mitotic division, while the zygote remains in a dormant period; thus, its division occurs later than that of the primary endosperm nucleus. The primary endosperm nucleus divides rapidly, undergoing nuclear division without the formation of cell walls. This results in the formation of a large number of free endosperm nuclei within the embryo sac. These nuclei are similar in size, most being mononucleate, though some may be binucleate or trinucleate (Figure 8a). They have not yet formed cell walls and are surrounded only by dense cytoplasm (Figure 8b). The newly formed endosperm nuclei are located at the center of the embryo sac. As the central vacuole forms and the number of free endosperm nuclei increases, the nuclei develop along the embryo sac wall from the micropyle end toward the chalazal end. A thin layer is formed between the embryo sac wall and the central vacuole membrane, with the nuclei connected by cytoplasm in a bead-like arrangement (Figure 8c). This structure gradually disappears as the endosperm develops (Figure 8d).

### 3.5. Degeneration of the Embryo Sac

The degeneration of the embryo sac in *T. taishanensis* is a key issue commonly encountered during its reproductive development (Figure 8e,f). This degeneration is characterized by complete coverage across all developmental stages, a variety of degeneration forms, and a high incidence during the mature stage, which severely restricts seed formation and population reproduction. Observations using resin semi-thin sections revealed that embryo sac degeneration is not limited to specific developmental stages. It occurs to varying degrees throughout the entire developmental process, from the binucleate embryo sac, tetranucleate embryo sac, to the mature embryo sac. Degenerative phenomena were most frequently observed in the mature embryo sac stage, which may represent one of the important contributing factors associated with a high empty-seed rate of up to 90% in *T. taishanensis*.

Degenerating cells exhibit characteristics such as deepening of the stain and cytoplasmic condensation, with cell structures gradually disintegrating. The degeneration can be classified based on the site of onset, including types where the egg apparatus degenerates first, the central and synergid cells degenerate synchronously, or the antipodal cells disintegrate prematurely, leading to systemic decay. This degeneration is random, occurring at different stages in different ovules within the same ovary, or at different developmental stages in the same ovule. It is particularly concentrated at the mature embryo sac stage, with some ovules degenerating shortly after fertilization or even before fertilization. Potential causes for this degeneration include insufficient or low vitality of the pollen supply, abnormal pollen tube germination or growth, developmental defects in the embryo sac itself, as well as environmental factors such as temperature fluctuations and changes in humidity during the flowering period.

The high frequency of embryo sac degeneration observed in sections is closely associated with an extremely low embryo formation rate in *T. taishanensis*. In section observations, it is difficult to obtain complete embryo structures at different developmental stages. Specifically, due to the high degeneration rate of embryo sacs, clear micrographs of intermediate developmental stages—including binucleate and tetranucleate embryo sacs and early proembryo stages—were rarely obtainable. Most ovules examined in sections exhibited degenerating embryo sacs rather than normally developing ones. This limitation in image documentation directly reflects the extremely low embryo formation rate of this species, which underlies its poor seed germination capacity.

## 4. Discussion

### 4.1. Characteristics of the Temporal Sequence and Synchrony in the Development of Male and Female Reproductive Organs

The developmental pattern of *T. taishanensis* is similar to that of other *Tilia* species such as *Tilia miqueliana*, whereas their developmental temporal sequences differ significantly [17]. The differentiation of male gametophytes initiates in late May, and mature bicellular pollen grains form in late June. By contrast, the development of female gametophytes starts in early June, with mature eight-nucleate embryo sacs formed in late June. Thus, the initiation of female gametophyte development is delayed by 1–2 weeks compared with male gametophytes, although both gametophytes reach maturity synchronously in late June (Table 1). This reflects temporal asynchrony in gametophyte development. However, such developmental-level asynchrony cannot be considered functional protandry, because pollen release and stigma receptivity were not monitored in the present study.

The flowering period of *T. taishanensis* lasts only 15 days, and the asynchronous development of male and female gametophytes is a unique characteristic of this species. Theoretically, this trait can prolong the pollination duration. However, combined with the pollen viability rate of merely 10%, the asynchronization between the attenuation of pollen viability and the maturation period of female gametophytes increases the risk of fertilization failure [18]. Meanwhile, obvious asynchrony is observed during the meiosis of microspore mother cells in *T. taishanensis*. Specifically, the developmental difference ranges from 3 to 4 stages among different locules of a single anther, and reaches 4 to 6 stages among different anthers. Although this feature broadens the pollination window, it further exacerbates the shortage of viable pollen, thereby hindering the normal reproductive development of plants.

### 4.2. Correlation Between Structural Developmental Characteristics of Male and Female Reproductive Organs and Embryogenesis

The pollen morphology of *T. taishanensis* conforms to the typical characteristics of the genus *Tilia*. However, its pollen viability is merely 10%, and the uneven distribution of exine ornamentation impedes water absorption and germination of pollen grains. The anther wall develops according to the dicotyledonous type, and the secretory tapetum develops from uninucleate cells to binucleate cells to supply essential nutrients for microspore development. Nevertheless, abnormal degeneration timing occurs in partial tapetal tissues, which interrupts nutrient supply to microspores [19,20]. Combined with asynchronous meiosis of microspore mother cells [21,22], these defects collectively result in pollen abortion and insufficient pollen production, forming a “pollen bottleneck” that restricts fertilization.

The embryo sac of *T. taishanensis* belongs to the Polygonum type, and its ovule is anatropous, crassinucellate and bitegmic. The intermediate cells of T-shaped trichomes on the outer ovary wall degenerate during development, which may block nutrient translocation. The style is of the closed type, and the compatibility between the structural characteristics of transmitting tissue and pollen tube growth remains to be further verified [23]. In addition, integument development is asymmetric in some ovules, which may prevent pollen tubes from penetrating into the embryo sac and consequently interfere with fertilization progression [24]. In addition, the cavities containing lightly stained material observed in the style and ovary wall may represent secretory tissues. In the style, these cavities may produce extracellular matrix components that support pollen tube growth through the transmitting tract. In the ovary wall, they may secrete protective or nutritive substances for developing ovules.

The embryogenesis of *T. taishanensis* follows the Solanad type, and its endosperm develops in the nuclear type. This overall developmental pattern is consistent with the common traits of the genus *Tilia* and angiosperms, reflecting the typical developmental rules of angiosperm embryogenesis [25]. Nevertheless, the coordination between embryo and endosperm development is essential for normal embryo formation, and uncoordinated developmental rhythms between these two tissues may directly trigger embryo abortion.

### 4.3. Comprehensive Regulatory Mechanism of Seed Abortion

Multiple factors may contribute to seed abortion in *T. taishanensis,* including defective reproductive structures, asynchronous developmental schedules, potential environmental stresses and low genetic diversity. Low pollen fertility, abnormal tapetal degeneration and embryo sac degeneration together constitute a “triple reproductive barrier”. Among these factors, continuous embryo sac degeneration (degenerative phenomena were most frequently observed at the mature stage) may represent one important contributing factor associated with the high empty seed rate of approximately 90%, although a direct causal relationship between embryo sac degeneration and seed abortion has not been experimentally demonstrated in this study. Moreover, amphitropous ovules may further impede nutrient supply to embryo sacs.

From the perspective of developmental chronology, asynchronous development of male and female gametophytes shortens the fertilization window and reduces the probability of effective fertilization. Even if fertilization is accomplished, the developmental coordination disorder between embryo and endosperm [26] may trigger premature disintegration of endosperm [27], resulting in embryo abortion due to nutrient deprivation. In terms of external environmental factors, temperature fluctuations and humidity variations during the flowering period are potential environmental constraints affecting pollen development in *Tilia*, which can exacerbate inherent developmental defects and adversely affect pollen viability, pollen tube germination and embryo sac stability [28]. However, without in-situ monitoring data from the sampling year, we cannot confirm the actual impact of short-term microclimate changes on pollen abortion. The superposition of multiple factors creates a vicious cycle, which drastically reduces the seed reproductive capacity of this species.

### 4.4. Limitations of the Study

This study is limited by the fact that all experimental materials were derived from a single wild mature individual of *T. taishanensis*, the only confirmed adult wild individual of this narrowly endemic species. The embryological characteristics and reproductive defects reported here should therefore be interpreted as representative of this particular individual and local population rather than generalized to the entire species. Despite this constraint, these data provide a valuable baseline for understanding reproductive failure and guiding conservation of this extremely rare tree.

## 5. Conclusion

The flowering period of *T. taishanensis* lasts for 15 days, and the initiation of male gametophyte development takes place 1–2 weeks earlier than that of female gametophyte, showing obvious developmental-initiation asynchrony between gametophytes, although both gametophytes reach maturity synchronously in late June. Microspores ultimately develop into bicellular pollen grains, whereas megaspores differentiate into seven-celled, eight-nucleate embryo sacs of the Polygonum type. After fertilization, the embryo develops via the Solanad type and the endosperm follows the nuclear type, and division of the primary endosperm nucleus precedes that of the zygote. Nevertheless, asynchronous meiosis of microspore mother cells and abnormal tapetal development result in a pollen viability rate of only 10%, leading to insufficient supply of viable pollen. Meanwhile, embryo sac degeneration could be observed throughout the entire developmental process, and degenerative phenomena were most frequently observed at the mature embryo-sac stage. Combined with structural defects including degeneration of trichome cells on the outer ovary wall and asymmetric integument development, the fertilization success rate is further reduced. Collectively, asynchronous gametophyte development, abnormal reproductive structures, as well as genetic and potential environmental factors lead to a low embryo formation rate and poor seed reproduction capacity in this species. This study reveals the intrinsic reproductive characteristics of this endemic extremely small population, and provides an important theoretical foundation for subsequent conservation and artificial propagation of this species.

## Figures and Tables

**Table 1:** Comparison of megasporogenesis, microsporogenesis, and development of female and male gametophytes over time.

Development Time	Microsporogenesis, Development of Male Gametophytes	Megasporogenesis, Development of Female Gametophytes
25 May	Archesporial cells–Primary sporogenous cells–secondary sporogenous cells	Ovule primordia
7 June	Microsporocyte–Prophase I of microspore mother cell meiosis	Outer integument–Archesporial cells
13 June	Stages between prophase I of meiosis and tetrads	Inner integument
19 June	Uninucleate microspore	Megasporocyte and its meiotic division
24 June	Uninucleate microspore, binucleate microspore	Uninucleate embryo sac-2-nucleate embryo sac-4-nucleate embryo sac
27 June	Mature 2-celled pollen grains	Mature 8-nucleate embryo sac

## Data Availability

The original contributions presented in this study are included in the article. Further inquiries can be directed to the corresponding authors.

## References

[1] Liang S. (1985). Two New Species of *Tilia* from Shandong Province. Shandong For. Sci. Technol..

[2] Jurkšienė G., Danusevičius D., Kembrytė-Ilčiukienė R. (2023). Dendrological Secrets of the Pazaislis Monastery in Central Lithuania: DNA Markers and Morphology Reveal *Tilia × europaea* L. Hybrids of an Impressive Age. Plants.

[3] Manchester S.R. (1994). Inflorescence bracts of fossil and extant *Tilia* in North America, Europe, and Asia: Patterns of morphologic divergence and biogeographic history. Am. J. Bot..

[4] Hasanagić R., Ganguly S., Bajramović E., Hasanagić A. (2021). Mechanical properties changes in fir wood (*Abies* sp.), linden wood (*Tilia* sp.), and beech wood (*Fagus* sp.) subjected to various thermal modification process conditions. IOP Conf. Ser. Mater. Sci. Eng..

[5] Zheng G., Xia Y., Li S., Mo W., Guo J., Li J. (2021). Characterization of the complete chloroplast genome of basswood (*Tilia mongolica* maxim.). Mitochondrial DNA B Resour..

[6] Chen Z., Tao H., Peng Y., Huang J., Cheng Y., Tian W., Chang Y., Zhou Y. (2025). Amur linden honey and its principal polyphenols alleviate obesity and regulate gut microbiota in high-fat diet-induced mice. Food Chem. X.

[7] Cai K., Zhao Q., Li H., Zhang Q., Li Y., Han R., Jiang T., Pei X., Zhang L., Zhao X. (2023). Deciphering aroma formation during flowering in nectar tree (*Tilia amurensis*): Insights from integrated metabolome and transcriptome analysis. For. Res..

[8] Bao W., Shen Y., Verdonk J.C. (2024). Dynamic Changes in Flavonoids’ Accumulation Pattern in *Tilia miqueliana* Flowers at Different Developmental Stages Based on Widely Targeted Metabolomic Analysis. Forests.

[9] McCarthy D.M., Mason-Gamer R.J. (2019). Morphological Variation in North American *Tilia* and Its Value in Species Delineation. Int. J. Plant Sci..

[10] Li Q., Wang Z., Wang Y., Yan L.P., Liu C.L., Wang K.F., Xu J.G. (2018). Study on Grafting Propagation Technology of *Tilia* spp. J. Agron..

[11] Ming Y., Xin H., Wang H.L., Li W., Zhang F., Tang S. (2023). First Report of Leaf Spot Disease Caused by Alternaria alternata in *Tilia miqueliana* in Jiangsu Province, China. Plant Dis..

[12] Shi F., Cao Y., Gao Y., Qiu Y., Lu Y., Han B., Shen Y. (2024). The Impact of Magnetic Field and Gibberellin Treatment on the Release of Dormancy and Internal Nutrient Transformation in *Tilia miqueliana* Maxim. Seeds. Forests.

[13] Fujiwara T., Shimizu D., Kon K., Isshiki N., Tsunokuni H., Aoyagi S. (2000). A new method for detecting and localizing cell markers endocytosed by fibroblasts in epoxy resin semi-thin sections using scanning electron microscopy combined with energy dispersive X-ray microanalysis after ion-etching. J. Electron Microsc..

[14] Shimizu D., Fujiwara T., Kon K., Isshiki N., Tsunokuni H. (2001). Three-dimensional reconstruction by scanning electron microscopy from serial epoxy resin semi-thin sections after ion-etching. J. Electron Microsc..

[15] Wang X., Chen J., Hu L., Zhang J., Xiao F., Zhang S., Shao F., Huang L. (2023). Embryological Observations on Seed Abortion in Hibiscus syriacus L. and Physiological Studies on Nutrients, Enzyme Activity and Endogenous Hormones. BMC Plant Biol..

[16] Qiu Q., Tian X., Wu G., Wu J., Yuan D., Fan X. (2025). Basis of single-seed formation in chestnut: Cytomorphological observations reveal ovule developmental patterns of Castanea henryi. PeerJ.

[17] Gao F., Shen Y. (2010). Megasporogenesis, Microsporogenesis and Development of Male and Female Gametophytes in *Tilia miqueliana*. China For. Sci. Technol..

[18] Shen J., Shen Y., Li W., Shang Y., Ding J., Li R. (2007). Studies on Pollen Viability and Fertilization of Clivia miniata Regel. Acta Hortic. Sin..

[19] Zhang C., Zhang M., Zhu Q. (2014). Cytological observation of pollen development in ‘Ougan’(*Citrus suavissima* Hort. ex Tanaka) and its seedless mutant. J. Fruit Sci..

[20] Han A., Yin K., Song L., Xin H., Liu J. (2004). Cytological study of male sterility and pollen abortion in pear (*Pyrus* spp.) variety “Xinli 7”. J. Southwest Agric. Univ..

[21] Jiang H.-B., Yang S.-M., Liu Y.-F., Tian Y.-P., Sun Y.-N., Chen L.-B., Tang Y.-C. (2020). Biological characteristics and cytological studies on anther abortion of male sterile Camellia crassocolumna. Acta Agron. Sin..

[22] Ekici N. (2025). Anther structure and pollen development in Jurinea kilaea Azn. (Asteraceae). Caryologia.

[23] González V.V., Zini L.M., Ortega-Baes P., Ferrucci M.S. (2023). Gynoecium structure and pollen tube pathway in the cactus family with emphasis on tribe Trichocereeae (Cactaceae: Cactoideae). Bot. J. Linn. Soc..

[24] Broz A.K., Bedinger P.A. (2021). Pollen-Pistil Interactions as Reproductive Barriers. Annu. Rev. Plant Biol..

[25] Lee Y.-I., Lee N., Yeung E.C., Chung M.-C. (2005). Embryo Development of Cypripedium formosanum in Relation to Seed Germination In Vitro. J. Am. Soc. Hortic. Sci..

[26] Grini P.E., Jürgens G., Hülskamp M. (2002). Embryo and Endosperm Development Is Disrupted in the Female Gametophytic capulet Mutants of Arabidopsis. Genetics.

[27] Hojsgaard D., Greilhuber J., Pellino M., Paun O., Sharbel T.F., Hörandl E. (2014). Emergence of apospory and bypass of meiosis via apomixis after sexual hybridisation and polyploidisation. New Phytol..

[28] Boavida L.C., Vieira A.M., Becker J.D., Feijo J.A. (2005). Gametophyte interaction and sexual reproduction: How plants make a zygote. Int. J. Dev. Biol..

